# Time-Course Changes in Urine Metabolic Profiles of Rats Following 90-Day Exposure to Propoxur

**DOI:** 10.1038/s41598-019-52787-1

**Published:** 2019-11-18

**Authors:** Yu-Jie Liang, Pan Wang, Hui-Ping Wang, Ding-Xin Long, Ying-Jian Sun, Yi-Jun Wu

**Affiliations:** 1 Department of Veterinary Medicine and Animal Science, Beijing University of Agriculture, Beijing, 102206 P.R. China; 2 Laboratory of Molecular Toxicology, State Key Laboratory of Integrated Management of Pest Insects and Rodents, Institute of Zoology, Chinese Academy of Sciences, 1-5 Beichenxilu Road, Beijing, 100101 P.R. China

**Keywords:** Diagnostic markers, Animal physiology

## Abstract

As a major kind of carbamate insecticide, propoxur plays an important role in agriculture, veterinary medicine, and public health. The acute toxicity of propoxur is mainly neurotoxicity due to the inhibition of cholinesterase. However, little is known regarding the toxicity of propoxur upon long-term exposure at low dose. In this study, Wistar rats were orally administrated with low dose (4.25 mg/kg body weight/day) for consecutive 90 days. And the urine samples in rats treated with propoxur for 30, 60, and 90 days were collected and analyzed by employing ^1^H NMR-based metabolomics approach. We found that propoxur caused significant changes in the urine metabolites, including taurine, creatinine, citrate, succinate, dimethylamine, and trimethylamine-*N*-oxide. And the alteration of the metabolites was getting more difference compared with that of the control as the exposure time extending. The present study not only indicated that the changed metabolites could be used as biomarkers of propoxur-induced toxicity but also suggested that the time-course alteration of the urine metabolomic profiles could reflect the progressive development of the toxicity following propoxur exposure.

## Introduction

Propoxur is a widely used carbamate insecticide, which plays an important role in agriculture, veterinary medicine, and public health. Propoxur is mainly used for pest control and malaria eradication programs^1^. However, the excessive use of propoxur causes severe acute and chronic toxicity in non-target species including humans, which is hazardous for human health^2^, albeit the beneficial effect of propoxur use in increase of agricultural productivity and reduction of insect-borne diseases.

As a carbamate, propoxur could reversibly inhibit acetylcholinesterase activity^3,4^. In experimental animals, even 1/10 LD_50_ of propoxur prominently inhibited cholinesterase activity and disrupted functions of nervous systems^5^, although the cholinesterase activity could return to normal within several hours^6^. The symptoms of propoxur intoxication in humans are those of typical cholinergic crisis, which include vomiting, diarrhea, abdominal pain, salivation, blurred vision, profuse sweating, and temporary paralysis of the extremities^7^. To understand the toxicity of propoxur due to long-term low-dose environmental exposure, time course study is necessary. In this study, we investigated the use of metabolomics to study the subchronic toxicity of the carbamate insecticide.

Metabolomics is the quantitative measurement of the metabolic response of organisms to pathological stimuli^8^. It provides better opportunity for assessment of the toxicity of environmental stressors^9^, and human diseases diagnosis^10,11^. Nuclear magnetic resonance (NMR) spectroscopy is a widely used metabolomic analytical platform with simple and cost-effective yet retaining high sensitivity and specificity characteristics^12^. Using the data processing and statistical analysis such as principal components analysis (PCA) and partial least squares methods^13,14^, the NMR data of biofluids can reveal changes of a large number of endogenous metabolites^15^.

^1^H NMR spectroscopy-based metabolomic analyses of urine and serum of rats have been successfully used to study the toxicity of various xenobiotics such as heavy metals^16–19^ and pesticides such as organophosphorus compounds tri-phenyl phosphate and tri-butyl phosphate^20–22^ and pesticide mixtures^23–26^.

In this current study, the ^1^H NMR analysis together with multivariate pattern recognition (PR) techniques were used for the time course study of propoxur toxicity in rat. This approach of studying propoxur toxicity will identify metabolic alteration in different stages of the insecticide toxicity, and potential biomarkers of propoxur toxicity at different exposure stages.

## Results

### Changes in body weight and organ weight

**Figure 1:**
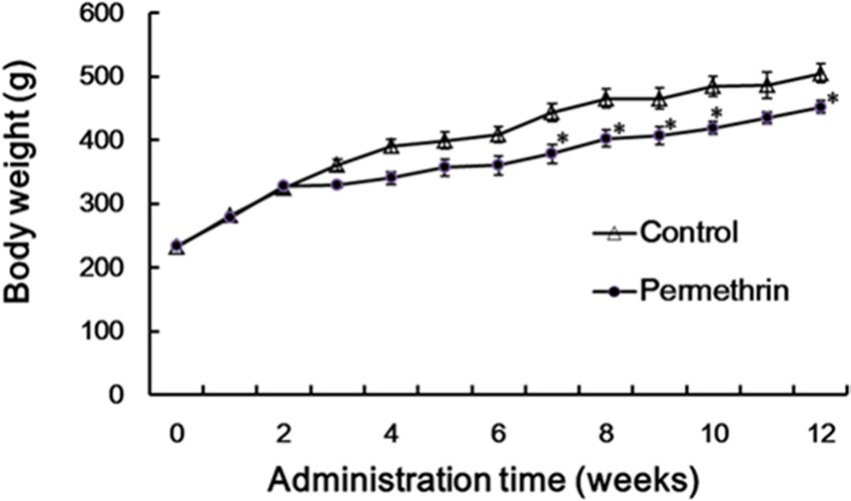
Change of the rat body weight following exposure to propoxur. The adult rats were orally dosed daily with propoxur (4.25 mg/kg body weight/day) for consecutive 90 days and the body weight of each rat was recorded daily. Data were expressed as mean ± SE with n = 5. ^*^P < 0.05, compared with the control at the same time point.

No obvious toxic signs were observed in the propoxur-treated rats. However, the body weight of the propoxur-treated rats decreased significantly compared with the control after the 90-day subchronic exposure (Fig. 1). The weight of organs was measured and the data was shown in Table 1. We found that only liver weight was decreased significantly after 90-day exposure; however, no obvious difference in all other organ weights was observed between the propoxur-treated rats and the control rats (Table 1).

**Table 1:** Organ coefficients of rats after 90-day exposure of propoxur.

Relative organ weights	Dose level (mg/kg body weight/day)	
	0	4.25
Terminal body weight (g)	479.7 ± 13.3	439.7 ± 8.1^*^
Brain (g%)	0.437 ± 0.027	0.481 ± 0.017
Heart (g%)	0.275 ± 0.010	0.271 ± 0.026
Liver (g%)	2.94 ± 0.17	2.33 ± 0.09^**^
Spleen (g%)	0.202 ± 0.021	0.199 ± 0.027
Kidneys (g%)	0.667 ± 0.073	0.668 ± 0.064
Testis (g%)	0.861 ± 0.062	0.894 ± 0.048
Adrenals (mg%)	16.2 ± 3.7	15.7 ± 1.5

Note: ^*^P < 0.05 and ^**^ P < 0.01, n = 5, compared with the control (0 mg/kg body weight/day).

### Effect of propoxur on clinical biochemistry and histopathology of liver and kidneys

**Figure 2:**
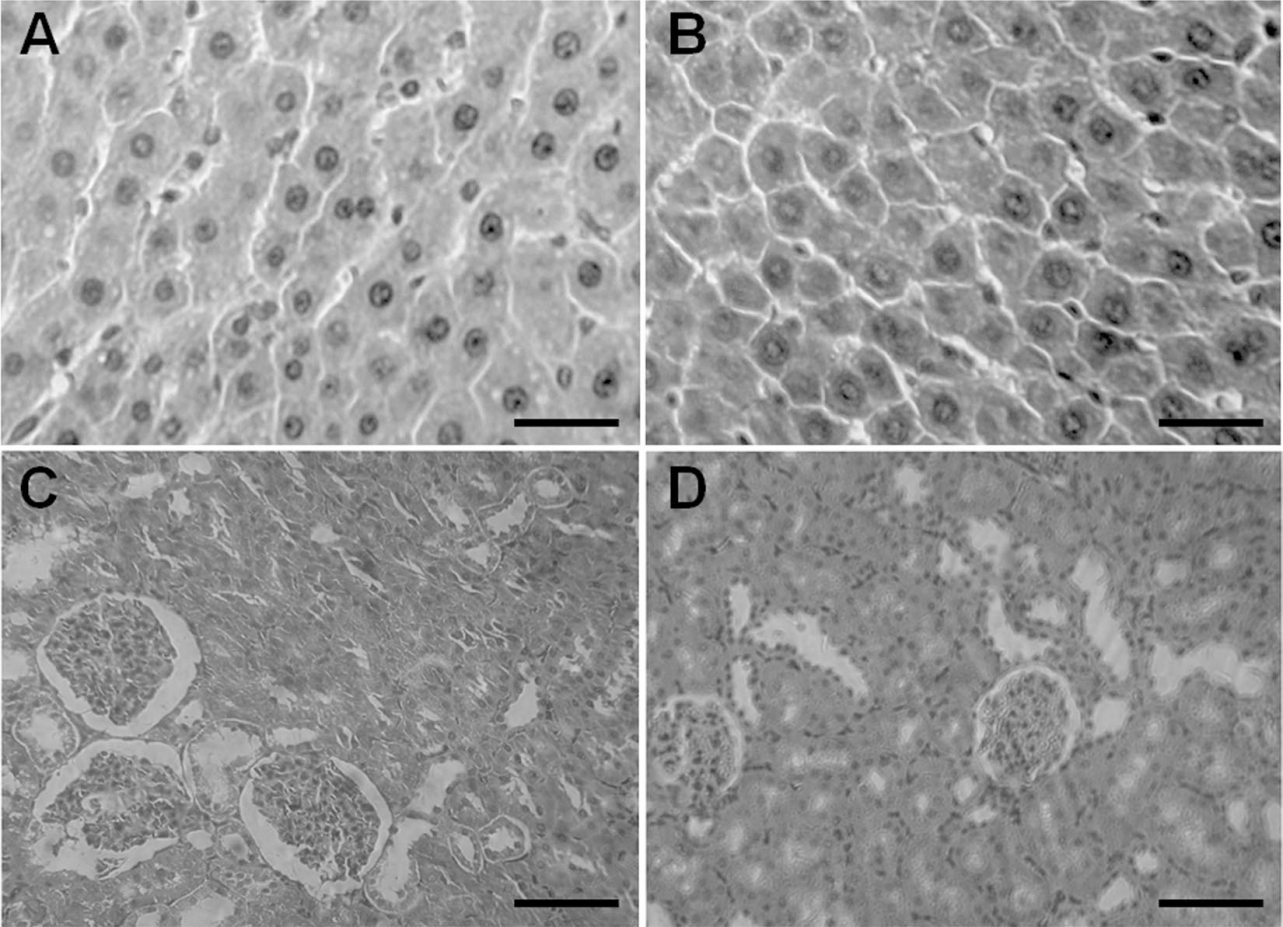
Photomicrographs of representative sections of the liver and kidneys of rats. Control rats demonstrate normal structure of hepatocytes (**A**) and nephridial tissue (**C**). Rats treated with propoxur (4.25 mg/kg body weight/day) for 90 consecutive days exhibited prominent swollen hyperchromatic nuclei (**B**) and a normal kidney histological structure (**D**). Scale bar: 50 μm.

The changes of the biochemical parameters in serum of the rats after 90-day exposure of propoxur were shown in Table 2. We found that serum cholinesterase (ChE) activity was inhibited by 34% in the propoxur-treated rats compared with the vehicle-treated rats (Table 2). Pathological sections of liver and kidney tissues of the rats exposed to propoxur are shown in Fig. 2. Microscopy examination found that liver parenchymal cells from the propoxur-treated rats changed with prominent swollen hyperchromatic nuclei (Fig. 2B), suggesting lesser liver damage was induced upon 90-day exposure of propoxur. However, no obvious histopathological change associated with kidney damage was observed in the kidney sections (Fig. 2D), which may suggest propoxur at the dose used in this study could not induce the kidney damage.

**Table 2:** Serum biochemical parameters of rats after 90-day exposure of propoxur.

Parameters	Control	Propoxur
ChE (U/L)	350.75 ± 56.80	230.5 ± 49.44^*^
Cr (mg/dL)	45.00 ± 4.42	43.25 ± 4.79
BUN (mg/dL)	6.57 ± 1.51	6.65 ± 1.13

Note: ^*^P < 0.05, n = 5, compared with the control group. Abbreviations: ChE, cholinesterase; Cr, creatinine; BUN, blood urea nitrogen.

### NMR spectroscopy and pattern recognition analysis of urine

**Figure 3:**
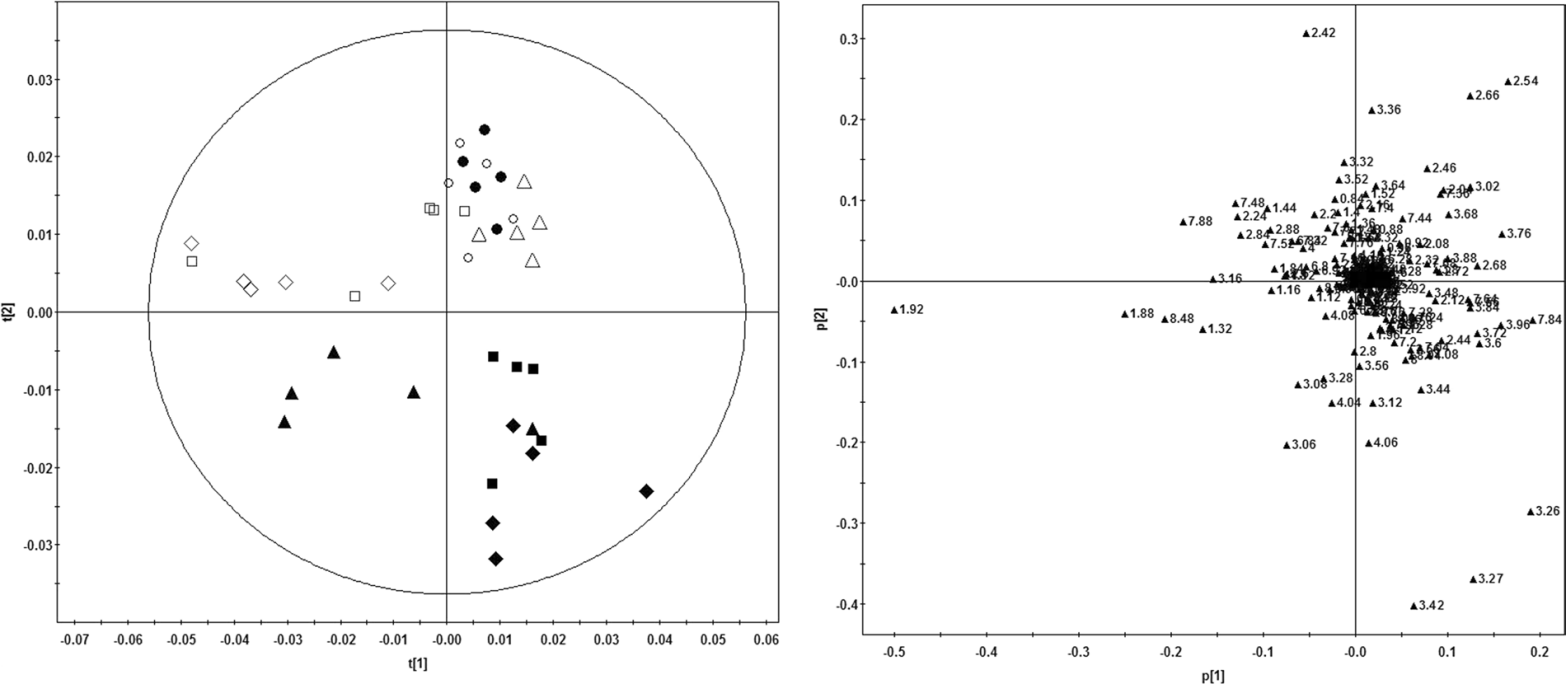
Principal component analysis (PCA) based on urine ^1^H NMR spectra obtained from control rats and propoxur-treated rats (4.25 mg/kg body weight/day for 90 days) at four time points including 0 day. (**A**) PCA score plot (PC1/PC2) for all samples. (**B**) PCA loading plot revealing the spectral regions (variables) responsible for the discrimination. Key: control group - 0 day (⚪), 30 days (△), 60 days (□), and 90 days (◊); propoxur group - 0 day (●), 30 days (▲), 60 days (■), and 90 days (♦).

Figure 3 shows the results of PCA analysis of urine spectrometry from the rats. Each point in the score plot represents an individual sample from each rat. It was found that the dots from control rats at different time points were all in the upper half of the circle and mainly grouped in the upper right area except all those from 90-day time point and some from the 60-day time point, which were in the upper left area. However, the dots from the treated rats were all in the lower half of the circle and mainly in the lower right area while only some dots at the 30-day time point are in the lower left area, except those at 0-day point, which were all in the upper right area and almost overlap with those from the control rats at the same time point. From this figure we found that at the very beginning of the experiment, there was no obvious difference in metabolomics profiles between the control rats and the treated rats. However, in the propoxur-treated rats, after longer exposure to propoxur, the metabolomics profiles changed greatly and the dots of treated groups separated gradually from those of the control groups, although the metabolomics profiles also changed slightly in the control rats especially after 60 days, which may suggest the normal biochemical changes due to physiological development of the test animals. Nevertheless, there was seldom overlapping of the dots from the treated rats in the four sampling time points (Fig. 3A).

The corresponding loading plot showed which metabolites contributed most to the separation of samples in the score plots. In the PCA loading plot, most dots, which represent the metabolites, are mainly around the zero points, while only a few distributed apart from the zero point, which determined the difference between the treatment groups (Fig. 3B). The time-course alteration of metabolomic profiles of the treated rats reflects the development of the propoxur toxicity. Thus, the PCA score plot revealed that propoxur time-dependently affected the urinary metabolite profiles.

**Figure 4:**
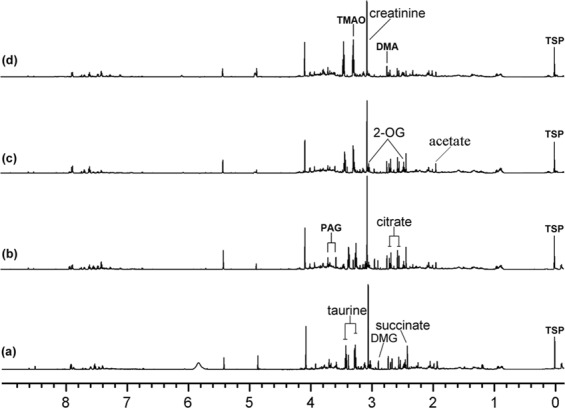
600 MHz ^1^H NMR spectra (δ 0.5–9.5) of urine in rats treated with propoxur (4.25 mg/kg body weight/day) for 0 day (**a**), 30 days (**b**), 60 days (**c**), and 90 days (**d**). Abbreviations: 2-OG, 2-oxoglutarate; DMA, dimethylamine; DMG, dimethylglycine; PAG, phenylacetylglutamine; TMAO, trimethylamine-N-oxide.

Figure 4 showed the typical NMR spectrums in each treatment group. There is no obvious difference of the metabolomic profiles of the rats at the first sampling time point after the beginning of propoxur administration (the 30th day after the first dosing) between the treated and the control except for citrate (2.54, 2.66 ppm), succinate (2.42 ppm), and 2-oxoglutarate (2-OG, 2.46, 3.02 ppm), which are slightly lower in propoxur group compared with that in control (Fig. 4a,b). However, at the second sampling time point (the 60th day after the first dosing), alterations of the profiles were obviously in the urine were obvious. Propoxur caused an increase in taurine (3.26, 3.42 ppm), creatinine (3.06, 3.04 ppm), trimethylamine-N-oxide (TMAO, 3.27 ppm), and dimethylglycine (DMG, 2.94 ppm), while caused a decrease in citrate (2.54, 2.66 ppm), phenylacetylglutamine (PAG, 3.76 ppm), succinate (2.42 ppm), and 2-OG (2.46, 3.02 ppm) (Fig. 4a,c). All these altered metabolites had a more drastic change at the third sampling time point (the 90th day) compared with those at the second sampling time point (the 60th day) (Fig. 4a,d). The insecticide-induced perturbations in the metabolites of urine were summarized in Table 3.

**Table 3:** Summary of the changes of urine metabolites induced by propoxur.

Main metabolites	Chemical shifts (ppm)	Days after initial dosing		
		30	60	90
Taurine	3.26 (t)	=	↑	↑↑^**^
Creatinine	3.06 (s), 4.06 (s)	=	↑^*^	↑↑^*^
Citrate	2.54 (d), 2.66 (d)	↓	↓	↓↓^*^
2-OG	2.46 (t), 3.02 (t)	↓^*^	↓^*^	↓↓^**^
Succinate	2.42 (s)	↓	↓^*^	↓↓^**^
PAG	3.76 (m)	=	↓	↓↓^*^
DMG	2.94 (s)	=	↑	↑↑^**^
TMAO	3.27 (s)	=	↑	↑↑^*^
DMA	2.73 (s)	=	=	↑
Acetate	1.92 (s)	↓	↓^*^	↓↓^**^

Note: The rats were treated daily with propoxur (4.25 mg/kg body weight/day) for 90 consecutive days. Metabolite changes relative to controls are indicated by ↑, ↑↑, ↓, ↓↓, or = , which represents an increase, a further increase, a reduction, a further reduction, or no change, in metabolites, respectively. Keys: d, doublet; m: complex multiplet; s, singlet; t, triplet. Abbreviations: 2-OG, 2-oxoglutarate; TMAO, trimethylamine-N-oxide; DMA, dimethylamine; DMG, dimethylglycine; PAG, phenylacetylglutamine. ^*^P < 0.05 and ^**^P < 0.01, compared with the controls (n = 5).

## Discussion

In this study, we employed NMR-based metabolomics approach to reveal that the metabolomic profiles changed along with the treatment time of propoxur. We found that propoxur caused changes in metabolomic profiles in rat urine. Loading plots and spectra revealed metabolomic changes including the elevation of creatinine, taurine, etc. The elevation of urine taurine and creatinine has been found to be a biomarker for liver damage^27,28^. The liver histopathological examination showed that rat liver parenchymal cells had prominent swollen hyperchromatic nuclei, while kidney tissues displayed normal structure after 90-day exposure. It was noticed in our previous studies that the liver damage with hepatocellular necrosis and vacuolation after the rats were dosed with propoxur for 28 consecutive days at a higher dose (1/10 LD_50_) and slightly histopathological changes at a lower dose (1/25 LD_50_)^29,30^; however, kidney histopathological examination showed that no pathological change was found in the rats exposed to propoxur for 28 days even at high dose (1/5 LD_50_)^29^. We found that tricarboxylic acid (TCA) cycle intermediates or TCA-related metabolites including citrate, succinate, 2-OG, and acetate decreased in urine after propoxur treatment (Table 3), suggesting a reduced or slower catabolism in hepatocytes^31,32^. Thus, propoxur might affect the energy metabolism in liver.

Moreover, the decrease of citrate in urine is found to be a biomarker for renal tublular acidosis caused by renal tubular dysfunction^33^. While the increase of dimethylglycine (DMG) in urine usually suggested that the renal papillae was damaged^18^. Thus, propoxur could cause the damage in not only liver but also kidneys although no abnormal structure was oberved in the histological sections of kidneys by regular microscopic examination at that time point. Actually, the metabolomics profile analysis of urine samples not only revealed the toxicity of propoxur in the organs but also provided information on the mechanism of its toxicity.

Our previous study reported that compared to histopathology and clinical chemistry, ^1^H NMR-based metabolomics was much more sensitive in detecting organ toxicity^25^. Consistently, in this study, the ^1^H NMR analysis is found to be able to detect the change of the metabolites as biomarkers, and with the time extension of the exposure, more metabolites were found to be changed in the levels and bigger alteration of levels of the metabolites were observed, which could predict the toxicities (e.g. hepatotoxicity and nephrotoxicity) of the insecticide at the different stages of the exposure.

In sum, the present study revealed that propoxur caused prominent changes of the urine metabolomics in rats. In addition, we identified the urine biomarkers for the propoxur exposure. This analysis of time course-based urine metabolomics profiling is a useful non-invasive *in vivo* assay for the toxicity of pesticides following long-term exposure.

## Materials and Methods

### Chemicals

Propoxur (2-isopropoxyphenyl N-methylcarbamate) (purity >97%) was kindly provided by the Hunan Research Institute of Chemical Industry (Changsha, China). 2,2′,3,3′-deuterotrimethylsilylproprionic acid (TSP), D_2_O, and other chemicals were obtained from Sigma Chemical Co. (St. Louis, MO, USA).

### Animals and treatment

Male Wistar rats with average weight of 200 ± 20 g were purchased from Weitong Lihua Laboratory Animal Technology Company (Beijing, China) and were housed individually in stainless steel cages. Animals were acclimatized for at least 1 week before the commencement of the study. During the experiment, the environmentally controlled conditions (room temperature: at 22 ± 2 °C and 50–60% humidity) and a light/dark cycle of 12 h were maintained. Animals had free access to water and commercially prepared laboratory animal diet. All animal procedures were performed strictly in accordance with current China legislation and approved by the Institute of Zoology Animal and Medical Ethics Committee.

Ten rats were randomly divided into 2 groups with 5 animals in each group. Previous studies showed the acute oral half-lethal dose (LD_50_) of propoxur was 85.1 mg/kg for male rats^34^. In this study we chose the doses of 1/20 LD_50_ (4.25 mg/kg body weight/day) of the pesticide as the dose for the pesticide treatment group.

The pesticide was dissolved in corn oil (1 ml/kg body weight for rats) and administered via oral gavage. The rats in control group received an equivalent volume of corn oil. Rats were given pesticide daily for 90 consecutive days. The body weight of each rat was recorded daily throughout the experimental period. Behavior and survival were monitored daily after dosing.

### Sample preparation

During the experiment, at the points of 30 days, 60 days, and 90 days from the first administration, 24-hour urine samples of each rat were collected into ice-cold vessel containing 1% sodium azide (0.1 ml) to prevent bacterial contamination. The urine samples then were centrifuged at 3000 × g for 10 min to remove any particulate matter, after which an aliquot was taken from each sample and stored at −80 °C until NMR analysis.

Twenty-four hours after the final administration, all rats were anesthetized and decapitated. Blood samples were collected and then centrifuged. The serum was collected for clinical biochemistry assays. Organ weights were immediately measured.

### Histopathology

Liver and kidney samples were fixed in 10% formalin overnight. The tissues were dehydrated in a graded series of alcohol, cleared in xylene, and then embedded in paraffin. Sections were cut with 4-µm thickness, and then rehydrated and stained with hematoxylin and eosin. The slides were observed with microscope (Olympus, Tokyo, Japan).

### Serum clinical biochemistry

Biochemical parameters of serum samples were analyzed on an Autolab-PM4000 Automatic Analyzer (AMS Co., Rome, Italy). The values of the parameters were expressed as mean ± SD.

### ^1^H-NMR spectroscopic measurement of urine samples

Urine samples were thawed, and an aliquot urine sample (400 μl) were homogenized with 200 μl phosphate buffer (0.2 M Na_2_HPO_4_/0.2 M NaH_2_PO_4_, pH 7.4) to minimize chemical shift variation due to different pH in urine samples. The urine-buffer mixture was let it on stand for 10 min and then centrifuged at 3500 × g for 5 min to remove any precipitates. An aliquot of 500 μl supernatant was transferred into a 5 mm conventional NMR tube supplied with 50 μl of TSP dissolved in D_2_O solution (1 mM, final concentration). The TSP acted as the internal chemical shift reference (δ 0.0), whereas D_2_O was used for deuterium lock signal for NMR spectrometer. Urine analysis was acquired on a Bruker-Av600 spectrometer (Bruker Co., Germany) at 298 K. Water signals were suppressed by presaturation. ^1^H-NMR spectra were carried out at 30 °C, with an excitation pulse of 90°, and a relaxation delay of 5 seconds. This resulted in an acquisition time of 0.91 sec and give a total of 64 K data points. For processing the FIDs were subjected to exponential function equivalent leading to an additional 0.3 Hz line-broadening factor prior to Fourier transformation (FT).

### Data reduction of ^1^H NMR spectra

Using MestReC (version 4.8.1.1, Mestrelab Research, A Coruna, Spain), each ^1^H NMR spectrum was automatically phased, baseline corrected and segmented range in size from 0 to 10 parts per million (ppm) in the spectral region, and each ^1^H NMR spectrum was divided with δ 0.04 ppm. For the urine data, the water and urea region was removed (δ 4.2–6.0) prior to PR analysis, and the spectra were normalized to the total urine volume collected at each time point, to correct the variation in concentration.

### Pattern recognition of the ^1^H NMR spectra

The reduced data described above were scaled to the total integral of each spectrum before PR analysis. Statistical analysis was processed with the soft independent modeling of class analogy (SIMCA) software package (Version 11.5, Umetrics AB, Umeå, Sweden). The unsupervised pattern recognition method principal component analysis (PCA) was performed to examine the dominant intrinsic variation in the dataset^35,36^.

### Statistical analysis

One-way ANOVA and Student’s *t*-test was used to assess the statistical significance of differences in measured parameters between the two groups. *P* < 0.05 was considered statistically significant.
